# Gender Differences in Behavioral and Neural Responses to Unfairness Under Social Pressure

**DOI:** 10.1038/s41598-017-13790-6

**Published:** 2017-10-18

**Authors:** Li Zheng, Reipeng Ning, Lin Li, Chunli Wei, Xuemei Cheng, Chu Zhou, Xiuyan Guo

**Affiliations:** 1 School of Psychology and Cognitive Science, East China Normal University, Shanghai, China; 2 Shanghai Key Laboratory of Magnetic Resonance, East China Normal University, Shanghai, China; 3 Key Laboratory of Brain Functional Genomics, Ministry of Education, Shanghai Key Laboratory of Brain Functional Genomics, East China Normal University, Shanghai, China; 4 National Demonstration Center for Experimental Psychology Education, East China Normal University, Shanghai, China; 5 Department of Physics, East China Normal University, Shanghai, China; 6 College of Mechanical and Electrical Engineering, Beijing Polytechnic, Beijing, China; 7 Department of Psychology, Fudan University, Shanghai, China

## Abstract

Numerous studies have revealed the key role of social pressure on individuals’ decision-making processes. However, the impact of social pressure on unfairness-related decision-making processes remains unclear. In the present study, we investigated how social pressure modulated men’s and women’s responses in an ultimatum game. Twenty women and eighteen men played the ultimatum game as responders in the scanner, where fair and unfair offers were tendered by proposers acting alone (low pressure) or by proposers endorsed by three supporters (high pressure). Results showed that men rejected more, whereas women accepted more unfair offers in the high versus low pressure context. Neurally, pregenual anterior cingulate cortex activation in women positively predicted their acceptance rate difference between contexts. In men, stronger right anterior insula activation and increased connectivity between right anterior insula and dorsal anterior cingulate cortex were observed when they receiving unfair offers in the high than low pressure context. Furthermore, more bilateral anterior insula and left dorsolateral prefrontal cortex activations were found when men rejected (relative to accepted) unfair offers in the high than low pressure context. These findings highlighted gender differences in the modulation of behavioral and neural responses to unfairness by social pressure.

## Introduction

Fairness emerges as a key concept in social interactions and affects human behavior dramatically. Previous studies using the Ultimatum Game (UG) paradigm have investigated the impact of fairness considerations on human decision-making processes in a two-person bargaining process^1–6^. Typically, two players are involved in UG, one proposes how to split a sum of money (i.e., the proposer) and the other one decides to accept or reject the division schema (i.e., the responder). Given the fact that the responder’s acceptance leads to the suggested division of money and his/her rejection results in both parties empty-handed, the responder should accept all offers in a one-shot UG, according to standard economic models which idealize individuals as perfectly rational cognitive agents aiming to maximize personal benefits. However, it was revealed that responders were likely to reject extremely unfair offers to punish norm-violating behaviors^3,7^, which has been interpreted according to theories of inequity aversion^8^, reciprocity^9,10^, or negative emotion caused by perception of unfairness^11^.

A number of neuroimaging studies have explored the neural basis underlying the unfairness-related decision-making processes in the typical two-person UG. Several brain regions have been consistently observed, including anterior insula (AI), dorsal anterior cingulate cortex (dACC) and dorsolateral prefrontal cortex (DLPFC)^4,12–14^. Previous studies revealed a broad engagement of AI in encoding interoception and emotional experience^15^, uncertainty^16,17^, error awareness^18^, risk and risk prediction error^19,20^. AI activation observed in recent UG studies during receiving unfair offers has been proved to be associated with the detection of fairness norm violations^21,22^. For example, researchers found that AI activity correlated with both positive and negative norm prediction errors (i.e., a U-shape response), suggesting the role of anterior insula in encoding error signals associated with norm violations when individuals make financial decisions during social interactions^23^. The dACC was usually considered to be involved in detecting and monitoring response conflicts and errors^24–26^. A recent study found that when receiving 3:7 offers, increased dACC activation was found for individuals who expected a fairer distribution (that is, 4:6 and 5:5), suggesting the engagement of dACC in detecting conflicts related to social expectation violations^12^. In a series studies of Güroğlu *et al*.^13,27,28^, the responder received unfair offers in three different contexts, where receiving two coins (ten coins in total, i.e., 8/2 offer) is pitted against three alternatives offers: (i) 5/5 offer (fair-alternative), (ii) 2/8 offer (hyperfair-alternative) and (iii) 8/2 offer (no-alternative). AI and dACC were found to display a similar activation pattern, that is, they were more active in rejection of unfair offers when the proposer had no-alternative as well as acceptance of offers when the proposer had a fair- or hyperfair-alternative, indicating the role of both regions in detecting personal norm-violations. Taken together, these evidence consistently suggested the involvements of AI and dACC in responding to signals associated with norm violations. The DLPFC was found to be related to rejection of unfair offers in UG and its activation was thought to be associated with overriding self-interested impulses^1,13,14,29,30^. Recent studies also suggested that right DLPFC may play a key role in integrating information and selecting context-appropriate responses during the norm-based decision-making processes^31,32^. The activation of DLPFC observed in the UG paradigm could be also interpreted to be related to the integration of information and the selection of context-appropriate final decisions to unfair offers, with overriding self-interested impulses being one of the possible underlying processes.

It has been widely acknowledged that social pressure modulates decision-making processes. For example, when opinions or judgments of an individual differed from those supported by the majority, the individual is often inclined to give up his/her own opinions or judgments and conform to the majority^33,34^. It is worth noting that gender difference should be taken into account when considering the impact of social pressure on human behaviors. Compared to women, men tend to be less persuadable and conforming in social pressure contexts^35–37^, and they also show greater retaliatory aggressions when they confront social threat^38^. Empirical studies investigating gender differences in group discussions have reported that men generally exhibit a greater amount of disagreement with other person’s position and persist in their own opinion, whereas women typically engage in higher amounts of agreement and positive social behaviors^39^. Women are also considered to possess stronger interpersonal sensitivity and more concern about interpersonal harmony than men^40–42^. Thus, it could be speculated that there would be gender difference in responders’ reactions to unfair offers in social pressure contexts.

In the present study, we used a modified version of UG to manipulate social pressure by introducing a social pressure context for responders in which the division schema was not only decided by proposers but also supported by their friends. We predicted increased rejection rates for men responders and reduced rejection rates for women responders when encountering unfair offers in the high pressure relative to the low pressure context, given the well-documented gender differences in responding to pressure^36,38–42^. At the neural level, we expected the involvements of unfairness-related brain regions, such as AI, dACC and DLPFC in the modulation of social pressure on women and men responders’ reactions to unfair offers.

## Results

### Behavioral Results

**Figure 1:**
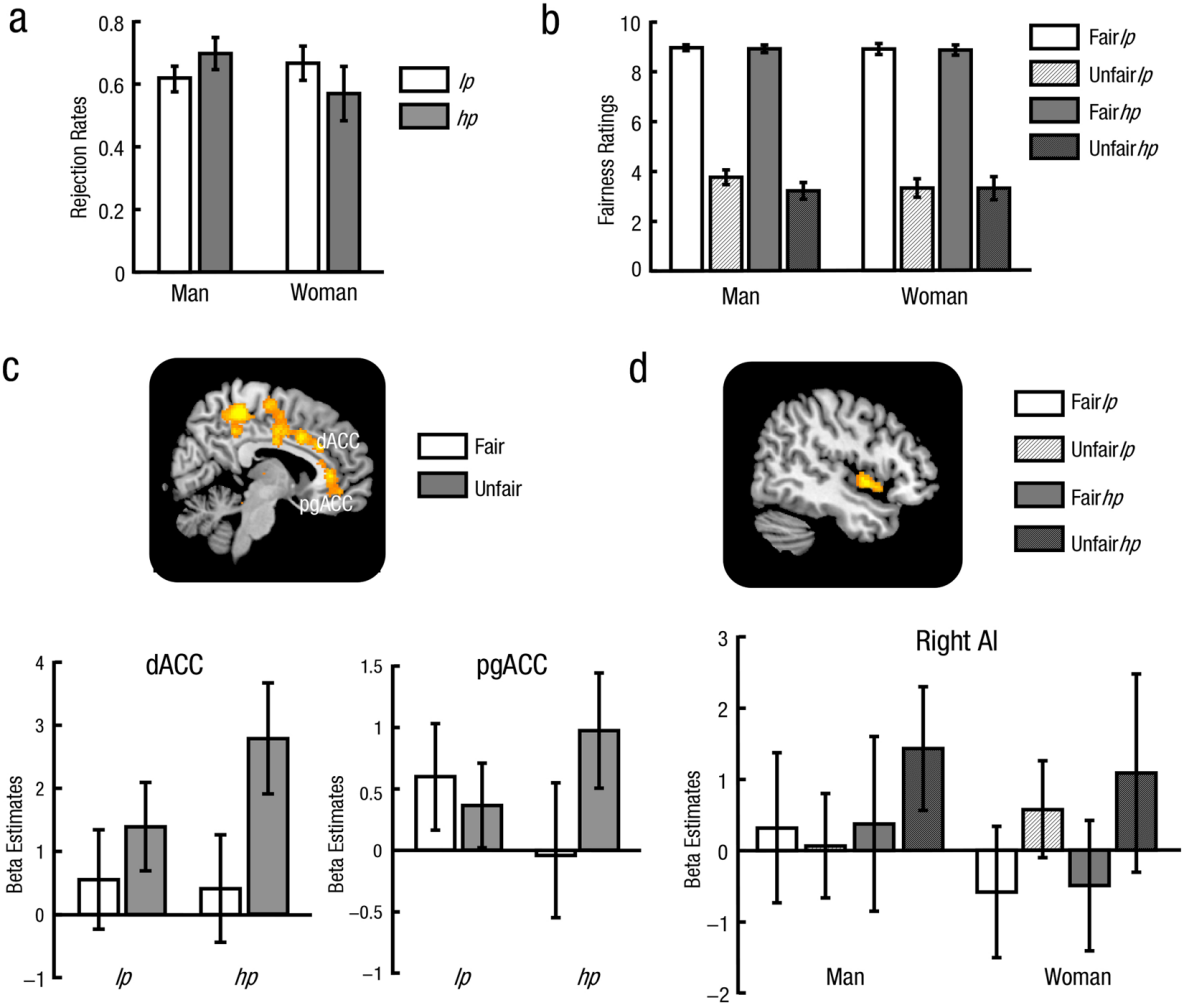
Behavioral results and unfairness-related effects in dACC and pgACC. Mean and 95% confidence intervals for rejection rates (**a**) and fairness ratings (**b**) in different conditions were showed. Women reported decreased rejection rates of unfair offers, whereas men reported increased rejection rates and decreased fairness ratings of unfair offers in the high pressure compared to the low pressure context. (**c**) Greater dACC and pgACC activations during unfair relative to fair trials were found in the high pressure compared to the low pressure context. (**d**) Unfairness-related activation in right AI was modulated by social pressure in men (but not in women). Error bars indicated 95% confidence intervals. Cluster level, *p* < 0.05, family-wise error corrected; voxel level, *p* < 0.001, uncorrected. pgACC = pregenual anterior cingulate cortex. dACC = dorsal anterior cingulate cortex. AI = anterior insula. *lp* = low pressure, *hp* = high pressure.

The rejection rates and fairness ratings (restricted to responded trials) for each participant in each condition were calculated (Fig. 1a and b). The average percentages of missed trials are 1.61% (and *SD* = 1.62%) in women and 0.93% (and *SD* = 1.07%) in men. Given that participants did not reject at all in the Fair conditions, a 2 (Gender: Woman vs. Man) × 2 (Context: High pressure vs. Low pressure) ANOVA on rejection rates was conducted only in unfair trials. Results revealed that there was no significant main effect of Gender (*p* = 0.187) or Context (*p* > 0.250), but a significant interaction (*F*(1, 35) = 9.96, *p* = 0.003, partial η^2^ = 0.22). *Post hoc* analyses showed that, compared to the low pressure context, rejection rates in the high pressure context was significantly larger in men (*t*(17) = 2.42, uncorrected *p* = 0.027, Cohen’s *d* = 0.57), but significantly smaller in women (*t*(18) = 2.18, uncorrected *p* = 0.043, Cohen’s *d* = 0.50). We also conducted another behavioral experiment with the same procedure to retest this effect. Results confirmed the dissociative effect of social pressure context on women’s and men’s rejection rates of unfair trials. See Supplemental Material available online for more details.

For fairness ratings, a 2 (Gender: Woman vs. Man) × 2 (Context: High pressure vs. Low pressure) × 2 (Unfairness: Unfair vs. Fair) ANOVA indicated significant main effects of Context (*F*(1, 35) = 5.81, *p* = 0.021, partial η^2^ = 0.14) and Unfairness (*F*(1, 35) = 2152.791, *p* < 0.001, partial η^2^ = 0.98). The main effect of Gender (*p* > 0.250), the two-way interaction between Context and Gender (*p* = 0.066) and the two-way interaction between Gender and Unfairness (*p* > 0.250) were not significant. More importantly, a significant two-way interaction between Context and Unfairness (*F*(1, 35) = 5.41, *p* = 0.026, partial η^2^ = 0.13) was found. *Post hoc* analyses showed decreased fairness ratings in unfair trials in the high pressure context compared to the low pressure context (*t*(36) = 2.46, uncorrected *p* = 0.019, Cohen’s *d* = 0.41), but not in fair trails (*p* > 0.250). A significant three-way interaction was also found (*F*(1, 35) = 7.12, *p* = 0.011, partial η^2^ = 0.17), indicated by a significant interaction between Context and Unfairness in men (*F*(1, 17) = 15.55, uncorrected *p* = 0.001, partial η^2^ = 0.48), but not in women (*p* > 0.250). Specifically, paired *t*-tests revealed significant difference between two contexts only in unfair trials (*t*(17) = 3.57, uncorrected *p* = 0.002, Cohen’s *d* = 0.84), but not in fair trials (*p* > 0.250) in men. No significant difference between two contexts was found in either fair or unfair trials in women (*p* > 0.250).

### fMRI Results

#### Interactions

$$\mathrm{Unfairness}-\mathrm{related}\,\mathrm{effects}:\,\mathrm{Context}\times \mathrm{Unfairness}\,{\rm{interaction}}$$

The Context × Unfairness interaction computed by the contrast (Unfair*hp* − Fair*hp*) − (Unfair*lp* − Fairn*lp*) revealed more activations in dACC (MNI –2 28 30) and pgACC (MNI 6 36 4) (Table 1). The opposite contrast found no significant activation. Beta values in different conditions were extracted from all the significant voxels in the 6mm-radius spherical regions centered on dACC (MNI –2 28 30) and pgACC (MNI 6 36 4) (beta values were extracted in the same way throughout the paper) and shown in Fig. 1c. It was found that greater unfairness-related activations (Unfair-Fair) in dACC and pgACC were observed in the high pressure compared with the low pressure context (dACC, *F*(1, 36) = 12.30, *p* = 0.001, partial η^2^ = 0.26; pgACC, *F*(1, 36) = 9.91, *p* = 0.003, partial η^2^ = 0.22).

$$\mathrm{Unfairness}-\mathrm{related}\,\mathrm{effects}:\,{\rm{Gender}}\,\times \,{\rm{Context}}\,\times \,{\rm{Unfairness}}\,{\rm{interaction}}$$

**Table 1:** Brain activations showing Context × Unfairness interaction and Context × Unfairness × Gender interaction.

Side	Region	Peak Activation			*t* Value	Voxels
		X	Y	Z		
**Context × Unfairness interaction**						
(Unfair*hp* − Fair*hp*) − (Unfair*lp* − Fair*lp*)						
L	Dorsal Anterior Cingulate Cortex	−2	28	30	4.23	1321
*R*	*Pregenual Anterior Cingulate Cortex*	*6*	*36*	*4*	*4.00*	
*R*	*Middle Cingulate Cortex*	*8*	*−8*	*36*	*3.86*	
R	Superior Temporal Gyrus/Rolandic Operculum	62	−16	12	4.85	2010
R	Superior Parietal Gyrus	14	−42	64	5.49	1932
L	Superior Temporal Gyrus	−64	−30	16	4.56	987
R	Middle Temporal Gyrus	50	−56	−2	4.4	287
(Unfair*lp* − Fair*lp*) − (Unfair*hp* − Fair*hp*)						
No Regions						
**Context × Unfairness × Gender interaction**						
Man[(Unfair*hp* − Fair*hp*) − (Unfair*lp* − Fair*lp*)] − Woman[(Unfair*hp* − Fair*hp*) − (Unfair*lp* − Fair*lp*)]						
R	Middle Insula	44	2	−2	4.32	215
*R*	*Anterior Insula*	*46*	*10*	*−6*	*3.84*	
Woman[(Unfair*hp* − Fair*hp*) − (Unfair*lp* − Fair*lp*)] − Man[(Unfair*hp* − Fair*hp*) − (Unfair*lp* − Fair*lp*)]						
No Regions						

Coordinates (mm) were in Montreal Neurological Institute (MNI) space. L = left hemisphere, R = right hemisphere. Cluster level, *p* < 0.05, family-wise error corrected; voxel level, *p* < 0.001, uncorrected. *lp* = low pressure, *hp* = high pressure.

There-way interaction between Gender, Context and Unfairness computed by the contrast Man[(Unfair*hp* − Fair*hp*) − (Unfair*lp* − Fairn*lp*)] − Woman[(Unfair*hp* − Fair*hp*) − (Unfair*lp *− Fairn*lp*)] revealed significant right AI activation (MNI 46 10 –6) (Table 1). The opposite contrast found no significant activation. As shown in Fig. 1d, in men, right AI was more active during unfair relative to fair trials in the high pressure context (*F*(1, 17) = 5.46, *p* = 0.032, partial η^2^ = 0.24), but not in the low pressure context (*p* > 0.250). However, right AI activity related to unfairness was not modulated by Context in women (*p* > 0.250).

$$\mathrm{Response}-\mathrm{related}\,{\rm{effects}}\,{\rm{during}}\,{\rm{unfair}}\,\mathrm{trials}:\,{\rm{Context}}\,\times \,{\rm{Response}}\,{\rm{interaction}}$$

To investigate the impact of context on responses to unfairness, we also tested the Context × Response interaction limited to unfair trials and only found significant right precuneus and right fusiform gyrus activations (Table 2).

$$\mathrm{Response}-\mathrm{related}\,{\rm{effects}}\,{\rm{during}}\,{\rm{unfair}}\,\mathrm{trials}:\,{\rm{Gender}}\,\times \,{\rm{Context}}\,\times \,{\rm{Response}}\,{\rm{interaction}}$$

**Table 2:** Brain activations showing Context × Response interaction and Context × Response × Gender interaction.

Side	Region	Peak Activation			*t* Value	Voxels
		X	Y	Z		
**Context × Response interaction**						
(UR*hp* − UA*hp*) − (UR*lp* − UA*lp*)						
No Regions						
(UR*lp* − UA*lp*) − (UR*hp* − UA*hp*)						
R	Precuneus	18	−52	22	5.29	1005
R	Fusiform Gyrus	38	−36	−14	4.77	281
**Context × Response × Gender interaction**						
Man[(UR*hp* − UA*hp*) − (UR*lp* − UA*lp*)] − Woman[(UR*hp* − UA*hp*) − (UR*lp* − UA*lp*)]						
L	Anterior Insula	−46	12	0	4.90	394
R	Anterior Insula	46	8	0	4.47	227
L	Dorsolateral Prefrontal Cortex	−32	52	22	4.55	371
L	Supplementary Motor Area	−2	6	54	4.38	472
R	Cerebellum	10	−70	−18	6.92	6868
R	Cerebellum	18	−40	−16	4.81	280
L	Cuneus	−4	−82	34	4.21	378
Woman[(UR*hp* − UA*hp*) − (UR*lp* − UA*lp*)] − Man[(UR*hp* − UA*hp*) − (UR*lp* − UA*lp*)]						
No Regions						

Coordinates (mm) were in Montreal Neurological Institute (MNI) space. L = left hemisphere, R = right hemisphere. Cluster level, *p* < 0.05, family-wise error corrected; voxel level, *p* < 0.001, uncorrected. UA = accepted unfair offers, UR = rejected unfair offers. *lp* = low pressure, *hp* = high pressure.

**Figure 2:**
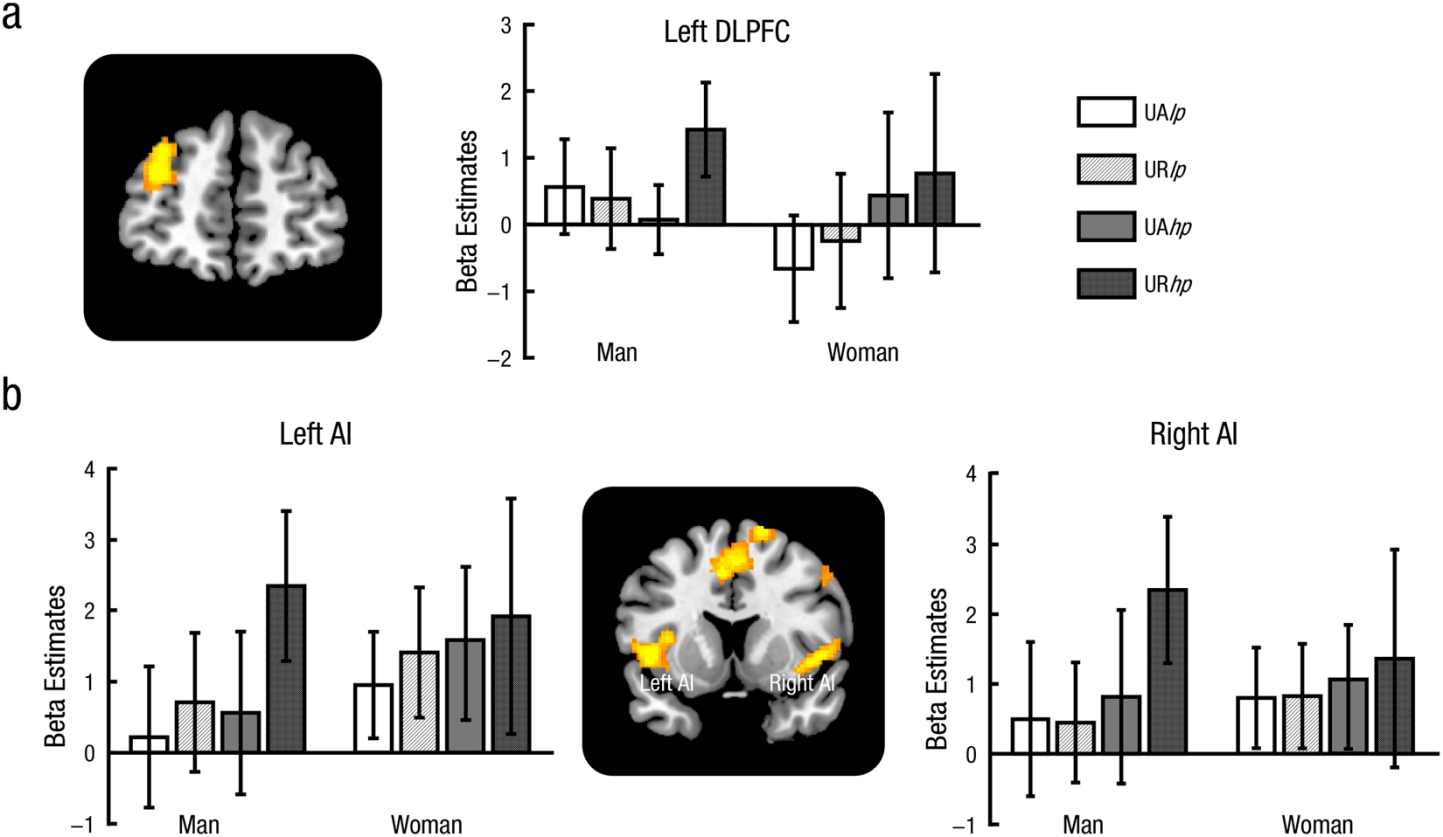
Response-related effects during unfair trials. Left DLPFC (**a**) and bilateral AI (**b**) activations during rejecting (relative to accepting) unfair offers were modulated by social pressure in men, whereas no significant interaction between Context and Response was found in women. Error bars indicated 95% confidence intervals. Cluster level, *p* < 0.05, family-wise error corrected; voxel level, *p* < 0.001, uncorrected. DLPFC =  dorsolateral prefrontal cortex. AI = anterior insula. UA = accepted unfair offers, UR = rejected unfair offers. *lp* = low pressure, *hp* = high pressure.

Significant activations in bilateral AI (MNI –46 12 0; 46 8 0) and left DLPFC (MNI –32 52 22) were observed in the contrast Man[(UR*hp* − UA*hp*) − (UR*lp* − UA*lp*)] − Woman[(UR*hp* − UA*hp*) − (UR*lp* − UA*lp*)]. We also computed the opposite contrast and found no activated region (Table 2). As shown in Fig. 2a and b, in men, bilateral AI and left DLPFC responded more strongly during rejecting relative to accepting unfair offers in the high pressure context (right AI, *F*(1, 17) = 11.95, *p* = 0.003, partial η^2^ = 0.41; left AI, *F*(1, 17) = 18.62, *p* < 0.001, partial η^2^ = 0.52; left DLPFC, *F*(1, 17) = 23.19, *p* < 0.001, partial η^2^ = 0.58), but not in the low pressure context (right AI, *p* > 0.250; left AI, *p* = 0.216; left DLPFC, *p* > 0.250). In women, no significant interaction between Context and Response was found (*ps* > 0.250).

#### Main effects

The main effect of Unfairness computed by the (Unfair - Fair) contrast revealed significant activations in supplementary motor area (MNI –4 20 48), and right AI (MNI 34 26 4). The reverse contrast revealed significant activation in right supramarginal gyrus (MNI 64–42 38). Contrasting trials in the high pressure context with trials in the low pressure context revealed significant activations in right calcarine gyrus (MNI 12 –86 0), right precentral gyrus (MNI 40 8 46), supplementary motor area (MNI –2 12 48), left hippocampus (MNI -22 –26 –6), and right thalamus (MNI 18–8 0). The reverse contrast showed no suprathreshold activation. The main effect of Gender defined by the (Man - Woman) contrast showed significant activation in left middle occipital gyrus (MNI -28 -78 30). The reverse contrast revealed no significant activation. Main effect of Response computed by the (UR - UA) contrast showed significant activations in left putamen (MNI -22 8 -2), right middle temporal gyrus (MNI 40-64 20), left superior parietal lobule (MNI -22 -64 44), bilateral precentral gyrus (MNI -32 0 46; MNI 66 6 20), and right fusiform gyrus (MNI 42-54 -16). The reverse contrast revealed no suprathreshold activation.

#### Correlation analysis

**Figure 3:**
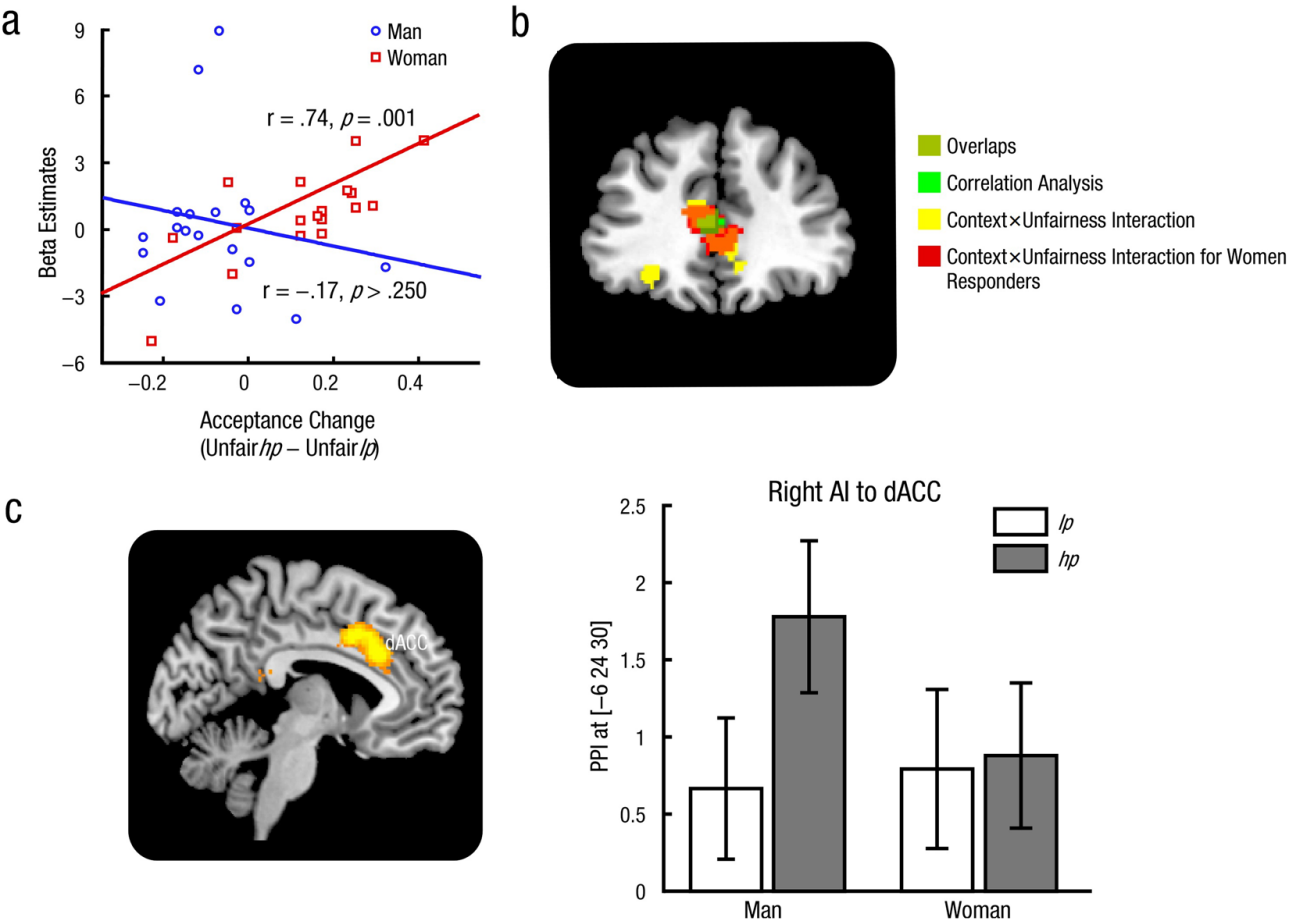
Results of correlation analysis and functional connectivity analysis. (**a**) The pgACC activity identified in the (UA*hp* − UA*lp*) contrast positively correlated with acceptance rate difference between the high pressure and the low pressure context in women. (**b**) The overlaps of pgACC identified in the correlation analysis, the Context × Unfairness interaction in general and the Context × Unfairness interaction in women were displayed. For display purpose, a voxel-level threshold of *p* < 0.001 (uncorrected) was used. (**c**) PPI analyses revealed significantly higher functional connectivity between right AI and dACC during unfair trials in the high pressure compared to the low pressure context in men. Error bars indicated 95% confidence intervals. Cluster level, *p* < 0.05, family-wise error corrected; voxel level, *p* <  < 0.001, uncorrected. pgACC = pregenual anterior cingulate cortex. dACC = dorsal anterior cingulate cortex. AI = anterior insula. *lp* = low pressure, *hp* = high pressure.

Brain-behavior correlation analyses revealed that a cluster located in pgACC (albeit only at the uncorrected level; MNI –2 40 12, cluster size = 73) identified in the (UA*hp* – UA*lp*) contrast positively correlated with corresponding behavioral acceptance rate difference between the high pressure context and the low pressure context in women (*r* = 0.74, *p* = 0.001, Fig. 3a). Interestingly, this pgACC overlapped with pgACC identified in the Context × Unfairness interaction. We further carried out two separate contrasts testing the Context × Unfairness interaction for women or men responders and found that pgACC (MNI 6 38 4) was significantly activated during the Context × Unfairness interaction in women, but not in men, though it did not survive the Gender × Context × Unfairness interaction. Figure 3b depicted the overlaps of pgACC activation in the correlation analysis, the Context × Unfairness interaction in general and the Context × Unfairness interaction in women.

#### Functional connectivity analysis

Whole-brain PPI analyses were performed for both women and men responders to examine how functional connectivity between right AI (MNI 34 26 4, coordinates were from the (Unfair – Fair) contrast) and other brain regions varied with social pressure in unfair trials. Results revealed that, right AI showed significantly higher functional connectivity with dACC (MNI –6 24 30) in the high pressure as compared to the low pressure context in men (Fig. 3c). No other significant modulation of functional connectivity was found.

## Discussion

The present study investigated how responders’ unfairness-related decision-making processes in the UG were modulated by social pressure. Results showed that women and men responders differed markedly in both behavioral and neural responses to unfairness in the high pressure context. Women reported reduced rejection rates, whereas men reported increased rejection rates and decreased fairness ratings of unfair offers in the high pressure relative to the low pressure context. Neurally, significant pgACC activation was found when women encountered unfair offers under social pressure. Activity difference in pgACC between accepted unfair trials with high pressure and accepted unfair trials with low pressure positively correlated with women’s acceptance rate difference between the two contexts. In men, stronger right AI activation and functional connectivity between right AI and dACC (here dACC could be specifically labeled as the sulcus ACC, for anatomical and functional dissociations between the sulcus and gyrus in ACC^43–45)^ revealed by the PPI analysis were observed during processing unfair offers in the high pressure relative to the low pressure context. Further analysis revealed that bilateral AI and left DLPFC were more active during rejecting relative to accepting unfair offers in the high pressure compared to the low pressure context.

Previous studies consistently revealed that, compared to women, men were less persuadable and conforming in social pressure contexts^35–37^ and exhibited greater retaliatory aggression when confronted with social threat^38^. These converging lines of evidence suggest that men are more likely to exhibit resistance behaviors towards social pressure, which was supported by the finding that men rejected unfair offers more often in the high pressure relative to the low pressure context in the present study. At the neural level, in men, stronger AI activation was observed in unfair offers relative to fair offers, and also in rejecting relative to accepting unfair offers in the high pressure as compared to the low pressure context. The PPI analysis also revealed higher functional connectivity between right AI and dACC during processing unfair offers, accompanied with decreased fairness ratings in the high pressure compared to the low pressure context. AI and dACC were not only related to detecting error signals associated with norm violations, but also served as two key regions of the salience network^12,13,21,23,46^. Taken together, our data suggested that men experienced more unfairness and detected more salient error signals related to fairness norm violations in the high pressure context, resulting in increased rejections of unfair offers, greater AI activation, and larger functional connectivity between AI and dACC during responding to unfairness.

Based on the widely accepted function of goal maintenance and executive control of DLPFC^47,48^, the DLPFC activation in the UG associated with rejection of unfair offers was interpreted to be related to the executive control of self-interested impulses^1,13,14,29,30^. An fMRI study using a trust game showed higher DLPFC activity for Machiavellians when their partner gave a cooperative offers in the previous round, which was interpreted to be associated with cognitive control of the reciprocal answer to the partner’s cooperative initiative to maximize their own benefits^49^. Recently, based on the results from their own studies and other evidence against a pure cognitive control model, Buckholtz *et al*.^31,32^ proposed a more comprehensive model named ‘integration-and-selection’ model to account for the function of right DLPFC in norm enforcement. This model suggested the critical role of right DLPFC in integrating information and selecting context-appropriate responses in norm enforcement. By using both UG and an impunity game (IG) in which the rejection could only reduce the responder’s income to zero, Cheng *et al*.^50^ further found greater left DLPFC activation when the responder rejected than accepted unfair offers in UG but not in IG, which was difficult to reconcile with the cognitive control model but was more in accordance with the ‘integration-and-selection’ model. In our dataset, men rejected more often and activated more left DLPFC during rejecting relative to accepting unfair offers in the high pressure compared to the low pressure context. Higher rejection rates for men may indicate more DLPFC activity engaged in controlling self-interested impulses^14,27,30–32^. Less conforming in social pressure contexts^35–37^ and greater retaliatory aggression towards social threat^38^ were observed for men relative to women. According to social role theory, men but not women learn that aggressive responding is an appropriate behavior that fit them better for the masculine role^51^. In this respect, rejection behaviors to revolt against proposers’ unfair treatments might be considered as an appropriate decision for men under social pressure, and the activation of DLPFC observed in the present study could be also interpreted to be related to selecting the context-appropriate rejection response to unfair offers^50^. Further evidence was needed to examine the validity of two models in accounting for the role of DLPFC in the UG studies in the future.

It was suggested that women possess stronger interpersonal sensitivity and concern about interpersonal harmony than men^40–42^. Empirical researches have revealed that women tend to be more persuadable and more conforming than men when confronted with social pressure^36,37^ and typically engage in high amounts of agreement and positive social behaviors during group discussions^39^. These findings suggest that women were sensitive to interpersonal threats and thus showed more compliance in the social pressure situations, which benefited the establishment and maintenance of interpersonal harmony. In the present study, women accepted unfair offers more often and showed increased pgACC activation during processing unfairness in the high pressure relative to the low pressure context. The behavior-brain correlation analysis further revealed that acceptance rate increase between two contexts positively related to activity in pgACC for women. These results indicated that pgACC may play a role in mediating women’s compliance behaviors under social pressure, consistent with the evidence that the pgACC/arMFC was engaged in perceiving and judging other people and mentalizing (especially during reading communicative intentions, but not private intentions of others)^52–54^. Nevertheless, the pgACC activity observed in our dataset was not an expected finding. Since that except for person perception and mentalizing, pgACC/arMFC was also associated with monitoring one’s own emotional state^52^, self-knowledge^52^ and conflict resolution^53^, which may be also possible explanations for the pgACC activation in the present study, future studies should be conducted to probe the exact function of pgACC during women accepting unfair offers under social pressure.

Additionally, superior and middle temporal gyrus were also found to be more activated when responders received unfair offers in the high pressure relative to the low pressure context. When focusing on the impact of social pressure on response-related effects in unfair trials, precuneus was significantly activated in the interaction between response and context. Further analysis revealed weaker activation in precuneus during accepting unfair offers in the low pressure context relative to the other three conditions (i.e., UR_*lp*_, UA_*hp*_, and UR_*hp*_). Prior studies have revealed the involvements of superior temporal gyrus, middle temporal gyrus, and/or precuneus in the process of mentalizing, that is, the ability to infer the other’s mental states^28,55–58^. It is possible that activations in precuneus, superior and middle temporal gyrus in the current study might be associated with understanding proposers’ intentions when responders received unfair treatments in the high pressure context and persuading themselves to accept unfair offers when these offers were not intolerant.

To conclude, these findings suggested that women and men differed significantly in behavioral and neural responses to unfairness in a social pressure context. More rejections of unfair offers in men and more acceptances of unfair offers in women were observed in the high pressure relative to low pressure context. Imaging data analyses suggested the role of pgACC in mediating women’s acceptance behaviors. In men, we observed higher functional connectivity between AI and dACC during processing unfairness and stronger recruitments of AI and DLPFC in the rejection responses to unfair offers in the social pressure context. Our data indicated the modulation of unfairness-related decision-making by social pressure and the gender difference in this process. In the future study, researchers should pay more attention to the generalization of the present findings to different social pressure contexts, different populations and different cultures.

## Method

### Participants

Thirty-eight right-handed volunteers [mean age = 22.79 years, *SD* = 2.81; 20 women and 18 men] from the university community with normal or corrected-to-normal vision participated in this experiment. None of the participants reported any abnormal neurological history. All the participants gave written informed consent before scanning. The data of one woman participant were excluded from further statistical analysis due to excessive head movements. The study was approved by the Ethics Committee on Human Experiments of East China Normal University. The methods were carried out in accordance to approved guidelines and regulations.

### Procedure

Before scanning, participants were told the rules of the game and that they would play as responders with many different proposers successively. They were then informed of the following information: all the offers about dividing money were collected before the experiment from real people; half of the offers were given by the proposers on their own (low pressure context), while the other half of the offers were collected from the proposers when they worked with three companions who supported their distributions (high pressure context); the decision regarding the offer from one proposer would not be disclosed to the other proposers; the offer of each trial was independent from other trials. In addition, participants were told that their payment for participating in the experiment depended on the outcome based on their decisions. However, in reality, participants were paid the amount of money obtained from a random selection of 5% trials in the game plus a 50 renminbi yuan (approximately equal to 32 dollars) bonus. In fact, there were no real proposers or supporters, and the offers were manipulated by the experimenter. The proposers and supporters in the task were represented by face pictures on the screen; One hundred and eighty neutral-expression face pictures of women and men from the Chinese Affective Face Picture System^59^, randomly allocated to serve as faces of proposers or supporters.

**Figure 4:**
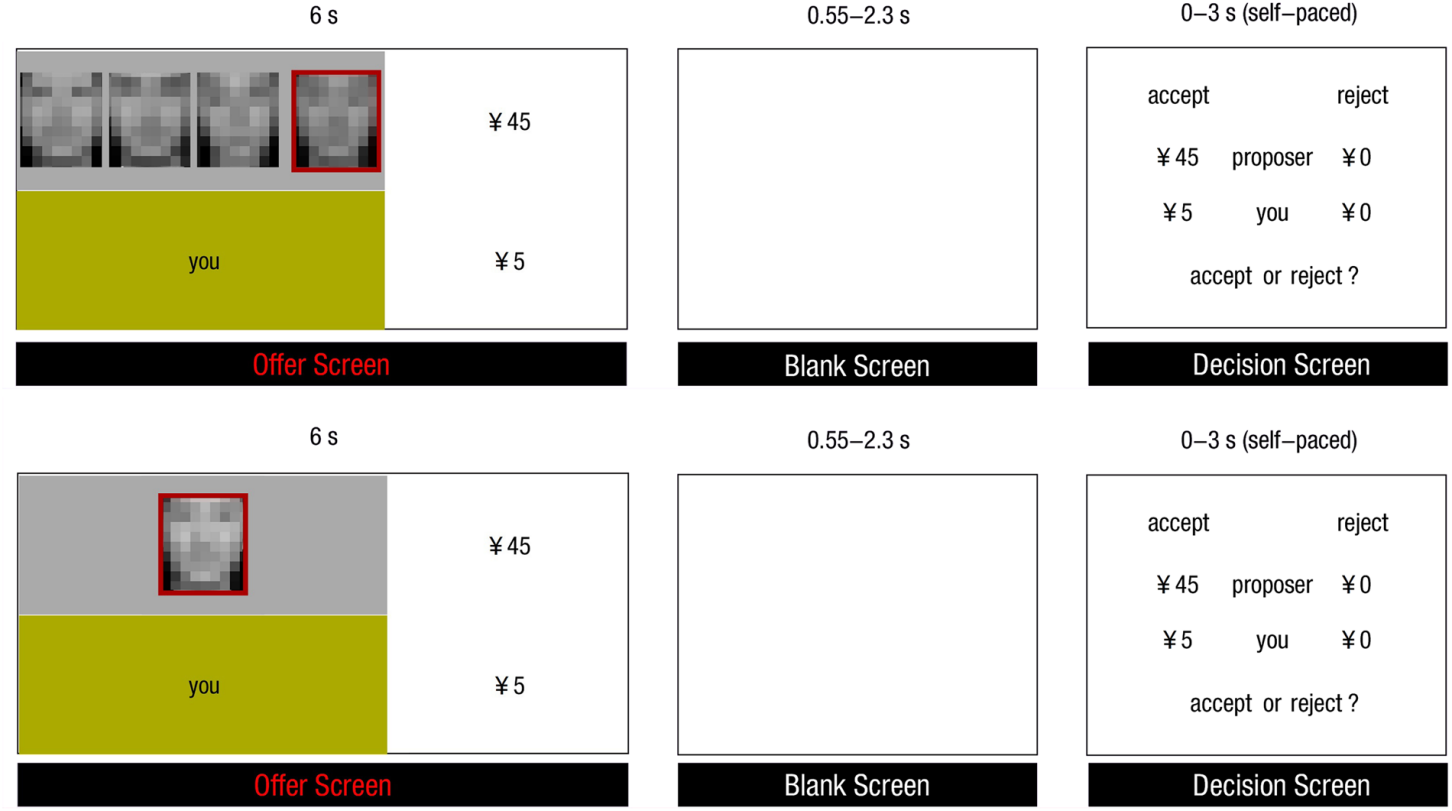
Experimental Procedure. The participant firstly received the offer from different proposer (marked by red borders) s in a context with low social pressure (lower) or in a high social pressure context (upper) in which the offer given by the proposer was supported by his/her friends. After a jittered blank lasting 0.55–2.3 s, the participant was asked to decide to accept or reject the offer within 3 s. During the experiment, participants would actually see faces without distortion, but not mosaic faces in this figure.

Participants then completed 72 trials in the scanner, 36 in each condition of social pressure context. Conditions were randomly intermixed and functional images were acquired simultaneously. As shown in Fig. 4, each trial began with a 6-s presentation of the offers given by the proposer. At the same time, the participant was informed whether the proposer had supporters by presenting the face of the proposer only or the faces of the proposer and his/her supporters. The faces of proposers in both contexts were marked by a red frame. Each condition of social pressure context contained 12 fair trials of 25:25 (i.e., a 25 vs. 25 distribution of 50) and 24 unfair trials, including 6 trials of 30:20, 6 trials of 35:15, 6 trials of 40:10 and 6 trials of 45:5. Afterwards, a blank screen jittering between 550 and 2300 ms was presented. After that, participants were asked to make a decision whether to accept or reject the offer within 3 s. Each trial was jittered with inter-stimulus intervals (approximately 3–8 s), during which a black fixation cross was presented. After scanning, the participants were presented with the same stimuli as inside the scanner and asked to rate how fair they felt for each offer using a 9-point Likert-type scale where 1 indicated extremely unfair and 9 indicated extremely fair.

### MRI Data Acquisition

Scanning was carried out on a 3.0-T Siemens scanner (Shanghai Key Laboratory of Magnetic Resonance, East China Normal University, Shanghai). Functional images were acquired using a gradient echo-planar imaging (EPI) sequence (repetition time = 2200 ms, echo time = 30 ms, field of view = 220 mm, matrix size = 64 × 64, 35 slices, slice thickness = 3 mm, gap = 0.3 mm). Before the functional run, a high-resolution structural image was acquired using a T1-weighted, multiplanar reconstruction sequence (MPR) (repetition time = 1900ms, echo time = 3.42 ms, 192 slices, slice thickness = 1 mm, field of view = 256 mm, matrix size = 256 × 256).

### Behavioral and Imaging Data Processing

For each participant, the rejection rates and fairness ratings were calculated for both fair and unfair trials in each context. Data were analyzed using mixed factors ANOVAs, with Context (High pressure vs. Low pressure) and Unfairness (Unfair vs. Fair) as within-subjects variables and Gender (Woman vs. Man) as a between-subjects factor. Though logistic regression implemented through generalized estimating equations (GEE) was also a valid statistical test, considering the common use of ANOVAs when analyzing behavioral data in the UG studies^13,22,30,60–63^, we adopted mixed factors ANOVAs to test rejection rates, which helped generate comparable results with previous studies. All the behavioral statistical analyses were performed using SPSS software (IBM SPSS Statistics for Windows, Version 23.0. Armonk, NY: IBM Corp.). It should be noted that, although 4:6 offers usually get the similar acceptance rate as 5:5 offers and are often included in the Fair condition^64,65^, many studies have revealed the significant decreased fairness ratings for 4:6 offers relative to 5:5 offers^1,5,66^. In the present study, offers of 25:25 were defined as the Fair condition and offers of 30:20, 35:15, 40:10, and 45:5 were defined as the Unfair condition, consistent with previous literatures^1,21,66,67^.

Imaging data preprocessing and statistical analyses were performed with Statistical Parametric Mapping (SPM8, http://www.fil.ion.ucl.ac.uk/spm/). During data preprocessing, the first five volumes were discarded to allow for T1 equilibration effects. The functional images were corrected for the delay in slice acquisition and were realigned to the first image to correct for interscan head movements. The individual structural image was co-registered to the mean EPI image generated after realignment. The co-registered structural image was then segmented into gray matter (GM), white matter (WM) and cerebrospinal fluid (CSF) using a unified segmentation algorithm^68^. The functional images after slice timing and realignment procedures were spatially normalized to the Montreal Neurological Institute (MNI) space (resampled at 2 × 2 × 2 mm^3^ voxels) using the normalization parameters estimated during unified segmentation and then spatially smoothed with a Gaussian kernel of 8 mm full-width half-maximum (FWHM).

Statistical analyses were performed using the general linear model (GLM) implemented in SPM8. At the first level analysis, two models were built. The first model accounted for gender difference in the impact of social pressure on unfairness-related effects. Thus four types of events (Fair*lp*: fair offers in the low pressure context, Unfair*lp*, unfair offers in the low pressure context; Fair*hp*: fair offers in the high pressure context, Unfair*hp*, unfair offers in the high pressure context) for women and men responders were separately conducted and included in this model. Events were convolved with a canonical hemodynamic response function (HRF). All the encoding trials were time-locked to the onset of the offers with null duration. Decision phase and trials with no response were also added into the model as additional covariates of no interest. Six regressors modeling the movement-related variance and one modeling the overall mean were also employed in the design matrix. High pass temporal filtering with a cutoff of 128 s was also applied in the models. For each participant at the first-level analysis, contrast images for each type of event were computed (Fair*lp*, Unfair*lp*, Fair*hp*, Unfair*hp*). At the second group level, these four first-level individual contrast images for women and men participants were fed into a 2 (Gender: Woman vs. Man) × 2 (Context: High pressure vs. Low pressure) × 2(Unfairness: Unfair, Fair) factorial design using a random-effects model (flexible factorial ANOVA in SPM8). In this ANOVA, we were interested in the modulation of unfairness-related effects by social pressure and gender differences during this process. Thus we tested for i) interactions between Context × Unfairness, ii) interactions between Gender × Context × Unfairness. Additionally, we also computed main effects of Gender, Context, and Unfairness.

To explore gender difference in the relationship between social pressure and responses to unfairness (rejection/acceptance), we built the second model in which unfair offers were further divided into accepted and rejected ones (UA*lp*, accepted unfair offers in the low pressure context, UR*lp*, rejected unfair offers in the low pressure context; UA*hp*, accepted unfair offers in the high pressure context, UR*hp*, rejected unfair offers in the high pressure context). The rest of the analysis was carried out in the same way as in the first model. In this model, one participant was excluded from further fMRI data analysis because of the lack of accepted unfair trials in the pressure context. Contrast images for four types of event (UA*lp*, UR*lp*, UA*hp*, UR*hp*) were computed for each participant at the first-level analysis and then fed into a 2 (Gender: Woman vs. Man) × 2 (Context: High pressure vs. Low pressure) × 2 (Response: UA, UR) flexible factorial ANOVA. In this ANOVA, we were interested in the impact of social pressure on response-related effects in unfair trials and gender differences during this process. Thus we tested for i) interactions between Context × Response, ii) interactions between Gender × Context × Response. Several whole-brain correlation analyses were performed separately for women and men responders to search regions whose activations detected from the (Unfair*hp* − Unfair*lp*), (UA*hp* − UA*lp*) or (UR*hp* − UR*lp*) contrast varied with behavioral changes between two contexts. Additionally, given that main effects of Gender and Context have been explored in the unfairness-related model which included all the valid trials, we only computed the main effect of Response in the response-related model.

We were also interested in how functional connectivity across brain regions during unfairness processing varied along different levels of social pressure in women and men responders. The psycho-physiological interaction (PPI)^69^ analysis was performed, with the individual time series from a 6-mm spherical region centered on the coordinates of right AI identified in the (Unfair – Fair) contrast of the second-level ANOVA as the physiological variable. In order to examine the context-dependent functional modulation of the connectivity pattern of right AI during unfair trials, a PPI model was conducted using a vector depending on the social context (1 for Unfair*hp*, –1 for Unfair*lp*) as the psychological variable. The PPI analysis was then carried out for each participant, resulting in the creation of a design matrix with the interaction term, the psychological variable and the physiological variable as regressors. Participant-specific contrast images of the interaction term were entered into a second-level random-effects analysis using a one-sample *t* test for women and men responders separately.

During data analyses, a cluster-level threshold of *p* < 0.05 (family-wise error corrected) and a voxel-level threshold of *p* < 0.001 (uncorrected) were used, unless otherwise indicated. The Anatomical Automated Labeling (AAL) atlas^70^ was used to identify activations with the MNI coordinates.

## Electronic supplementary material

Supplementary material
