# Supplementary material for: Gender Differences in Behavioral and Neural Responses to Unfairness Under Social Pressure
Source: Sci Rep. 2017 Oct 18;7:13498. doi: 10.1038/s41598-017-13790-6 (PMC5647364; doi:10.1038/s41598-017-13790-6)
Supplement: Supplementary file 1 — Supplementary material [file 41598_2017_13790_MOESM1_ESM.pdf]

Gender Differences in Behavioral and Neural Responses to Unfairness Under Social Pressure

Li Zheng[1, 2*], Reipeng Ning[3*], Lin Li[4], Chunli Wei[4], Xuemei Cheng[3], Chu Zhou[5, CA], Xiuyan Guo[6, 2, CA]

1. School of Psychology and Cognitive Science and Shanghai Key Laboratory of Magnetic Resonance, East China Normal University, Shanghai, 200062, China.
2. Key Laboratory of Brain Functional Genomics, Ministry of Education, Shanghai Key Laboratory of Brain Functional Genomics, East China Normal University, Shanghai, 200062, China.
3. Shanghai Key Laboratory of Magnetic Resonance and Department of Physics, East China Normal University, Shanghai, 200062, China.
4. School of Psychology and Cognitive Science, East China Normal University, Shanghai, 200062, China.
5. Department of Psychology, Fudan University, Shanghai, 200433, China.
6. Shanghai Key Laboratory of Magnetic Resonance and School of Psychology and Cognitive Science, East China Normal University, Shanghai, 200062, China.

CA. Corresponding Author: Chu Zhou & Xiuyan Guo

Chu Zhou E-mail: zhouchu@fudan.edu.cn Phone: +86 021-55665391

Xiuyan Guo E-mail: xyguo@psy.ecnu.edu.cn Phone: +86 021-62232908

*These authors contributed equally to this work.

Supplementary Material

A behavioral experiment with the same procedure as the fMRI experiment was conducted. Power calculations were performed to determine sample sizes by using the effect size obtained in the fMRI experiment (i.e. Cohen's $d = 0.50$ for women participants and Cohen's $d = 0.57$ for men participants). Sample sizes were determined as 44 for women participants and 35 for men participants based on a power of .9 and a Type I error level of .05 (two-tailed t-test). The 2 (Gender: Woman vs. Man) × 2 (Context: High pressure vs. Low pressure) repeated measures ANOVA on rejection rates of unfair offers revealed a significant interaction ($F(1, 77) = 10.59$, $p = .002$, partial $\eta^2 = .12$), indicated by increased rejection rates in men ($t(34) = 2.43$, $p = .020$, Cohen's $d = 0.41$) and decreased rejection rates in women ($t(43) = 2.30$, $p = .027$, Cohen's $d = 0.35$) in the high pressure context relative to the low pressure context (Figure S1a). For fairness ratings, a 2 (Gender: Woman vs. Man) × 2 (Context: High pressure vs. Low pressure) × 2 (Unfairness: Unfair vs. Fair) repeated measures ANOVA revealed a significant two-way interaction between Context and Unfairness ($F(1, 77) = 11.60$, $p = .001$, partial $\eta^2 = .13$), indicated by decreased fairness ratings in unfair trials in the high pressure context compared to the low pressure context ($t(78) = 4.72$, $p < .001$, Cohen's $d = 0.53$), but not in fair trials ($p > .250$). Though no significant three-way interaction was found, paired t-tests also revealed that significant difference for fairness ratings between two contexts was only found in men in unfair trials ($t(34) = 5.74$, $p < .001$, Cohen's $d = 0.97$), indicating that decreased men's fairness ratings in the high pressure context compared to the low pressure context may be a relatively robust effect (Figure S1b).

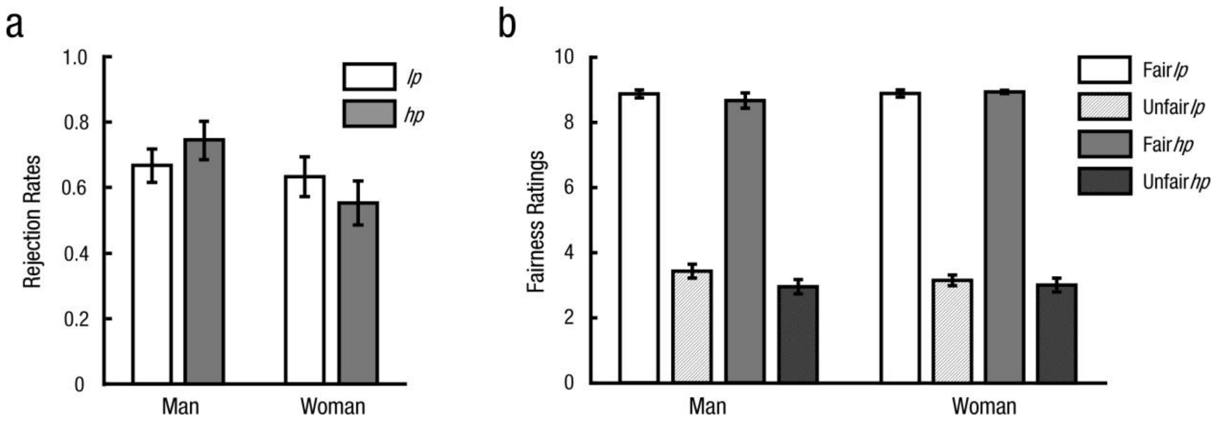

Figure S1 Mean and 95% confidence intervals for rejection rates (a) and fairness ratings (b) in different conditions were showed. Error bars indicated 95% confidence intervals. lp = low pressure, hp = high pressure.
